# Calciphylaxis Penile Lesion: An Unusual Complication of End-Stage Renal Disease

**DOI:** 10.7759/cureus.80625

**Published:** 2025-03-15

**Authors:** Julia Grote, Elizabeth Geyer-Roberts, Andrew Banuelos, Armand Edalati, Justin Ceballos

**Affiliations:** 1 Dr. Kiran C. Patel College of Osteopathic Medicine, Nova Southeastern University, Fort Lauderdale, USA; 2 Internal Medicine, Jackson Memorial Hospital, Miami, USA; 3 General Surgery, HCA (Hospital Corporation of America) Florida Westside Hospital, Plantation, USA

**Keywords:** complications related to calciphylaxis treatment, end stage renal disease (esrd), penile calciphylaxis, treatment of calciphylaxis, uremic calciphylaxis

## Abstract

Calciphylaxis is a rare disease that usually affects patients with end-stage renal disease (ESRD), also known as uremic calcific arteriolopathy. Calciphylaxis typically presents as an extremely painful skin nodule that progresses to ulcerated and frequently gangrenous lesions. Diagnosis can be difficult and treatment relies on a multidisciplinary approach to care. The following case describes a medically complex 37-year-old male patient with a painful penile calciphylaxis lesion. Calciphylaxis is a complex systemic disease that is difficult to diagnose. We highlighted in this case the importance of having a high index of suspicion in ESRD patients who present with a painful penile lesion.

## Introduction

Calciphylaxis, also known as uremic calcific arteriolopathy, is a rare systemic disease that commonly presents in patients with end-stage renal disease (ESRD). Although it can occur in patients without renal disease (termed non-uremic calciphylaxis), it most commonly presents as a complication of ESRD and affects 0.04-4% of ESRD patients [1]. Calciphylaxis is a devastating sequela associated with high morbidity and mortality rates [2]. The disease is characterized by microvascular calcification of medial arterioles, leading to narrowing and occlusion of vessels. As the disease progresses, worsening ischemia and endothelial damage cause the characteristic clinical manifestations of calciphylaxis. It presents initially as exquisitely painful cutaneous or subcutaneous lesions that appear as nodules or plaques. Lesions typically progress to become ulcerated, gangrenous, and frequently infected. Differential diagnoses include diabetic wounds, pyoderma gangrenosum, peripheral artery disease, or warfarin- or heparin-induced necrosis [3]. The diagnosis is typically made clinically, with suggested biopsy for patients with an atypical presentation. Treating calciphylaxis is primarily focused on controlling ESRD, providing symptom relief, risk factor reduction, and preventing further calcification and infection [1]. 

Calciphylaxis of the penis is extremely rare, with very few cases reported in the literature. Lesions are most commonly found on the lower extremities and in females [4]. Surgical management of penile calciphylaxis does not show an increased mean survival time compared to conservative measures; hence, conservative measures such as wound debridement and broad-spectrum antibiotics are often preferred [5]. However, one benefit of surgical management is that it has been shown to help with symptom management. The procedure of choice is a penectomy, where the necrosed area is excised. Removal is associated with reduced disease progression [5,6]. Additionally, a urinary diversion is also necessary in some patients as another form of symptom management. Patients often present with a gangrenous, pus-filled penis that may or may not include crepitus and signs of a necrotizing infection [5,6]. The penis may contain violaceous lesions or subcutaneous nodules and ulcerations. High mortality rates in these patients have led to little literature on follow-up data [5]. The following case describes an unusual presentation of a young male with a calciphylaxis penile lesion. 

## Case presentation

A 37-year-old male with ESRD on hemodialysis (HD) secondary to uncontrolled type 1 diabetes mellitus presented to our institution for a blackening lesion on the tip of his penis. At this time, the patient stated that he was currently receiving treatment for calciphylaxis and admitted that his current symptoms have been worsening, prompting him to be evaluated further. He reported worsening pain and redness around the lesion along with purulent drainage. The patient was also experiencing dysuria and urinary frequency. The patient has extensive additional medical history including congestive heart failure, hypertension, atrial fibrillation, and alcohol, cocaine, and marijuana abuse. 

**Figure 1:**
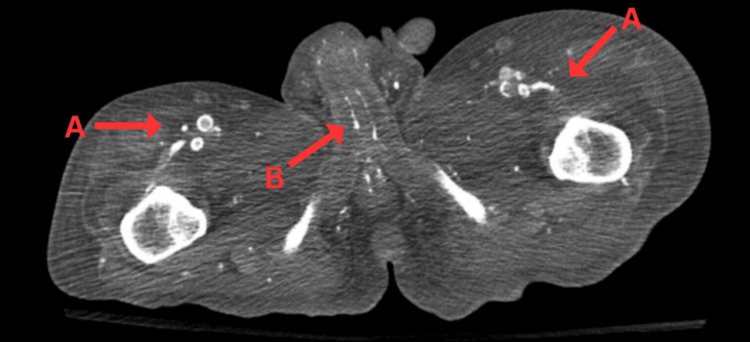
Axial view of abdominal/pelvic CT scan. The above figure demonstrates (A) diffuse calcification of the femoral artery and branches and (B) calcification of the penile terminal branches of the internal pudendal artery.

When the patient was evaluated, he appeared well and was in no apparent distress. He was found to have a black, gangrenous, and tender penile tip. Additionally, the patient also had dry and erythematous right metatarsal hallux necrosis and nonpalpable right posterior tibialis and dorsalis pedis pulses. An abdominal/pelvic CT scan was obtained and revealed diffuse calcification of the femoral artery and branches as well as calcification of the penile terminal branches of the internal pudendal artery (Figure 1). Femoral angiography was also obtained which demonstrated stenosis of the femoral artery.

During the course of his stay, HD was restarted via right tunneled catheter dialysis. Sodium thiosulfate was also resumed for the treatment of calciphylaxis. Urology was subsequently consulted and was determined that the patient did not warrant any surgical interventions for the penile wound. Additionally, wound cultures were performed, and grew extended-spectrumbeta-lactamase *Escherichia coli *(ESBL* E. coli*). He was then treated with meropenem, as recommended by the Infectious Disease consultants. Throughout the course of this patient’s hospital stay, local wound care was performed to the penile lesion. It was noted that the black eschar was progressively falling off and improving. Eventually, the patient was discharged to a skilled nursing facility.

## Discussion

Calciphylaxis is a rare condition affecting patients with ESRD. It is caused by the calcification of microvessels in the subcutaneous adipose tissue, leading to extremely painful ulcerated skin lesions. Risk factors for developing calciphylaxis include the following: female gender, ESRD, obesity, and diabetes. Derangements in calcium, phosphorus, and parathyroid hormone (PTH) may also contribute to the pathogenesis of this disease [1,2]. Patients diagnosed with calciphylaxis experience a one-year mortality rate of 45-80%, with infection of the ulcerated lesions being the main cause of death [2]. Penile calciphylaxis is an unusual finding as lesions typically present on the lower extremities and central areas of the body. This case study shows that physicians must have a high clinical suspicion when ESRD patients complain of a painful penis, as the pain may present prior to the onset of skin lesions. Diagnosis may be made clinically, but a biopsy may be helpful in distinguishing the lesion from other skin conditions. However, using biopsy in the diagnosis of calciphylaxis is controversial because it may worsen ulceration and increase the rate of infection [1,2]. Biopsies are contraindicated in patients with penile lesions, which may cause additional difficulty when diagnosing penile calciphylaxis [1].

The treatment of calciphylaxis is a multidisciplinary approach, yet remains debated. Primarily, pain control and adequate wound care must be initiated. The pain associated with calciphylaxis lesions is often refractory to high doses of opioids and might even require other medications such as gabapentin and ketamine. Wound care focuses on the removal of necrotic tissue and the prevention of local and systemic infections. Surgical debridement may be considered in patients with large or infected wounds; however, more long-term research is needed to know if it is associated with better survival rates [1,2]. Surgical debridement has been associated with increased symptom control, and in patients with penile calciphylaxis, penectomies and urinary diversions are the main options [5]. Penectomies involve the removal of the necrosed and gangrenous area of the penis. The urinary diversion reroutes the flow of urine to aid in symptom management. As calciphylaxis is such a deadly disease, follow-up data regarding these patients is limited. In a study of 50 cases of penile calciphylaxis, the mean time to death was 97 days from diagnosis. The average age was 54.5 years [6]. Extragenital gangrene was associated with an increased risk of death due to the involvement of additional blood vessels. For patients with increased involvement of blood vessels, various interventions are currently being explored such as revascularization surgery and balloon-expandable bare metal stent implantation of the arteries [6]. Additionally, hyperbaric oxygen therapy is also being studied as a potential wound care management of calciphylaxis, but it presents many limitations, such as cost and accessibility [7,8]. One of the most important treatment strategies in patients with penile calciphylaxis is risk factor control. Normal levels of calcium, phosphate, and PTH should be maintained and calcium supplements, vitamin D supplements, and warfarin should be avoided. Research into surgical parathyroidectomies is also being analyzed as a potential treatment to aid in controlling calcium, phosphate, and PTH levels [6]. 

Medications targeting the calcification of microvessels have also been used to treat calciphylaxis. Sodium thiosulfate is routinely used as an off-label treatment to prevent progression and improve symptomatology in patients with calciphylaxis. Sodium thiosulfate works as a calcium binder and antioxidant, which promotes the excretion of calcium in the urine or during dialysis while also protecting from inflammation and vasoconstriction [7-9]. Sodium thiosulfate is typically administered intravenously to patients during scheduled dialysis sessions at a dose of 25 mg [1,8,9]. The safety, efficacy, and duration of treatment are currently being studied in the clinical trials [1]. Some studies have proposed that supplementation of vitamin K may have the potential to treat calciphylaxis due to the ability to slow calcification of coronary arteries and aortic valve. One clinical trial is underway to study the effects of vitamin K supplementation in the treatment of calciphylaxis [1,7]. 

## Conclusions

This study presents the case of a young man with a rare presentation of calciphylaxis. It shows that lesions may occur in untypical areas, such as the genitals; hence, any presentation of erythematous purple skin, non-healing sores, or new urinary symptoms must be investigated thoroughly, especially in patients with ESRD. In conclusion, we highlighted the need for healthcare providers to have a high index of suspicion for calciphylaxis skin lesions since it can be frequently misdiagnosed as other skin conditions. Furthermore, this case also demonstrates the use of sodium thiosulfate and consistent wound care to manage penile calciphylaxis. In the future, there is a need for more extensive research on potential effective treatments and prevention of calciphylaxis.
